# Change of the Structural Properties of High-Performance Concretes Subjected to Thermal Effects

**DOI:** 10.3390/ma15165753

**Published:** 2022-08-20

**Authors:** Grzegorz Piotr Kaczmarczyk, Daniel Wałach, Eduardo Natividade-Jesus, Rui Ferreira

**Affiliations:** 1 Department of Geomechanics, Civil Engineering and Geotechnics, The Faculty of Civil Engineering and Resource Management, AGH University of Science and Technology, Al. Mickiewicza 30, 30-059 Cracow, Poland; 2 Department of Civil Engineering, Coimbra Polytechnic—ISEC, 3030-199 Coimbra, Portugal; 3 Institute for Systems Engineering and Computers of Coimbra (INESCC), 3030-290 Coimbra, Portugal

**Keywords:** internal structure, high-performance concrete, fibre-reinforced concrete, fire, computed tomography

## Abstract

The paper refers to studies of the structure of high-performance concrete with polypropylene fibre at different dosages. The authors see a research gap in the study of the effect of adding polypropylene fibre on the parameters of concrete exposed to high temperatures. The study takes into account the thermal effect—groups of samples were heated to 200 °C, 400 °C and 600 °C. The authors carried out basic tests to describe the changes in density, ultrasonic tests, uniaxial compression strength tests and tensile tests by splitting. The positive effect of polypropylene fibres is mainly observed between 20 °C and 200 °C. The melting of polypropylene fibres causes a delay in the development of micro-cracks in the structure of these concretes compared to HPC. Adding polypropylene fibres to the mixtures also increased the speed of ultrasonic wave propagation in the medium. The research was deepened with tomographic imaging. A description of the splitting surface was carried out. The results of tensile by splitting tests clearly show an increase in the relative failure area for unheated concretes in proportion to the number of fibres used. Changes in splitting surfaces under the influence of temperature are graphically illustrated. Furthermore, differences in the samples under the influence of heating at high temperatures are presented. Finally, the porosity development of all sample groups before and after heating at all temperatures is described.

## 1. Introduction

Human activity in the construction of ever larger and more complex projects is putting pressure on the development of technologies. These technologies should ensure minimal impact on the environment [1,2], propose solutions for improving the energy efficiency of buildings [3], reducing CO_2_ emissions using renewable energy [4,5], and utilise modern structural materials [6]. One of the most commonly used materials in construction is concrete [7] for which ongoing development of innovative research is observed, aimed at improving its design in obtaining appropriate properties and durability [8,9], and methods for the evaluation of achieved results [10]. Among the trends developed worldwide, the high-performance fibre-reinforced concrete (HPFRC) group is worth mentioning [11]. High-performance concrete (HPC) is characterised by a lower water–cement ratio and its main advantages are greater durability, higher compressive strength and better compactness [12,13,14]. It should be noted that under the selected conditions, the features resulting from lower porosity do not always give only positive results. The elements are characterised by a higher setting temperature and it can be difficult to properly cure the concrete. The lack of an extensive pore system makes it difficult for water to penetrate through the top layer of concrete, which makes HPC more susceptible to early-age cracking [15].

According to Eurocode 2 (PN-EN 1992-1-2:2008/NA:2010) [16], the compressive strength of concrete decreases with the increase of the exposure temperature, which relates to the higher strength class of the concrete [17]. This very general relationship has been confirmed by many studies, as can be found in papers [18,19,20].

HPC mixtures with polypropylene fibres that are intended for nuclear applications were tested for changes in mechanical properties after exposure to 200 °C [21]. Research conducted in [21] shows that the addition of polypropylene fibres (1.8 kg/m^3^) may lead to changes in residual compressive strength, modulus of elasticity and splitting tensile strength due to fibres melting during heating.

Research on the chemical changes in concretes themselves has already been carried out in the last century. There are numerous studies on the degradation of chemical compounds contained in concretes, which are cited by the authors of this paper. Transition and decomposition temperatures of cement phases are widely described in paper [22]. In the first phase, at a temperature of 50–100 °C, CaSO_4_·2H_2_O decomposes [23]. When heated to 100–150 °C, the further decomposition of CaSO_4_·2H_2_O has been reported in the literature [24] and the degradation of CaSO_4_·½H_2_O [25], Ca_3_Al_2_O_6_·3CaSO_4_·26H_2_O [23], CaAl_2_O_4_·10H_2_O [26] and Mg_6_Al_2_O_9_·CO_3_·12H_2_O [27] starts. After reaching a temperature of 150–200 °C, researchers indicate a further decomposition of [24] and CaSO_4_·½H_2_O [25]. It also mentions Ca_3_Al_2_O_6_·CaSO_4_·12H_2_O [28], Ca_2_Al_2_O_5_·8H_2_O [29] and Ca_4_Al_2_O_7_·13H_2_O [29].

Further weight losses occurred in samples heated to 400 °C. Concrete reaching temperatures in the range 200–250 °C loses further CaSO_4_·½H_2_O [23], Ca_2_Al_2_SiO_7_·8H_2_O and Al(OH)_3_ [29]. Al(OH)_3_ decomposes further up to 350 °C [29]. In addition, Ca_3_Al_2_O_6_·6H_2_O [23] and Mg_6_Al_2_O_9_CO_3_·12H_2_O [27] decompose at temperatures of 300–350 °C. When reaching 400 °C, CaSO_4_ [30] breaks down.

The next declines in chemical changes are recorded at 400–600 °C. Between 400 and 500 °C, the literature indicates that Mg(OH)_2_, Ca_3_Al_2_O_6_·CaSO_4_·12H_2_O, Ca_3_Al_2_O_6_·6H_2_O and Mg_6_Al_2_O_9_·CO_3_·12H_2_O breakdown occurs. One of the most important due to the loss of mass above 400 °C [22] is for the decomposition of Ca(OH)_2_ by the reaction Ca(OH)_2_ ➝ CaO + H_2_O ↑ [31]. Finally, at temperatures above 500 °C, Al(OH)_3_ [22], CaCO_3_ [23] and MgCO_3_ [22] are noted.

Due to the phenomena described, the authors see a research gap in the study of the effect of adding polypropylene fibre to the parameters of concrete exposed to high temperatures. The main area of research interest is the change in the structural behaviour of thermally exposed concrete. The authors wanted to understand how the compressive and tensile strength of concrete changes. In addition to the data obtained through strength tests, the authors decided to evaluate the internal structure and how it changes when exposed to high temperatures. In recent years, this topic has been addressed, among others, in the context of fire safety in building structures [32,33]. In this context, it will be particularly important in structures such as road tunnels, for which evacuation conditions can be difficult [34] and for which much higher demands are made on the high temperature resistance of concrete. In the research area, mainly plain concretes were analysed and only a few works dealt with the subject of high-performance concretes with the addition of polypropylene fibres, among others [17,35,36,37,38,39]. The results were not clear on the effect of fibre addition on the mechanical properties of concretes subjected to high temperatures, as well as the fact that they did not explain the change of the structural properties of high-performance concretes subjected to thermal effects. The different effects of a change in mechanical properties in relation to a temperature increase obtained by researchers may be due to the use of different types of aggregates [40]. The final values of the mechanical properties may also be affected by the curing conditions of the test samples [41].

In the study, the results of the basic research were compared with the literature data and X-ray tomographic imaging analysis was performed to provide an advanced description of the structural changes in the analysed concretes.

## 2. Materials and Methods

### 2.1. Preparation of Samples

The specimens were made from high-performance concrete under laboratory conditions. The concrete was prepared on the basic mix base shown in Table 1. During the preparation of the samples, mixes were also made with additional Texa-Fib 3 polypropylene fibres. Texa-Fib 3 fibres form a spatial mesh that functions as a micro-reinforcement for the concrete. According to the manufacturer, it is used for bridge concretes and heavy-duty industrial flooring. The manufacturer indicates a fibre density of 0.91 kg/L. The fibre thickness is 3.0 dtex with a length of 12 mm. The resulting specific surface area is, according to the manufacturer, ~0.22 g/m^2^. The fibres have a tensile strength of 420 N/mm^2^. From the point of view of the research problem, the deformation temperature reaching 145 °C is also important. The fibres are inserted into the mixer after adding the aggregate, but before adding the cement and liquid components (water and admixtures). According to the product sheet, Texa-Fib 3 consumption is 0.6 kg/m^3^.

The concrete mixtures were divided into four groups: HPC without additives, HPFRC 0.6 (concrete with 0.6 kg/m^3^ fibre), HPFRC 0.9 (concrete with 0.9 kg/m^3^ fibre) and HPFRC 1.1 (concrete with 1.1 kg/m^3^ fibre). A higher amount of superplasticiser was used in mixes containing polypropylene fibres, in order to produce a mix with rheological properties comparable to HPC. The mixtures were placed into 80 × 80 × 12 cm^3^ moulds and then compacted with a concrete vibrator. The blocks were cured for a period of 4 weeks. After the setting period, 50 mm diameter cylinders were cut from the slabs with a borehole drill. Each specimen underwent a process of grinding the surfaces of both bases to obtain parallel planes for compressive strength tests. The authors paid attention to the time function. The samples were stored at 20–25 °C for a period of three years.

### 2.2. Heating Process

**Figure 1:**
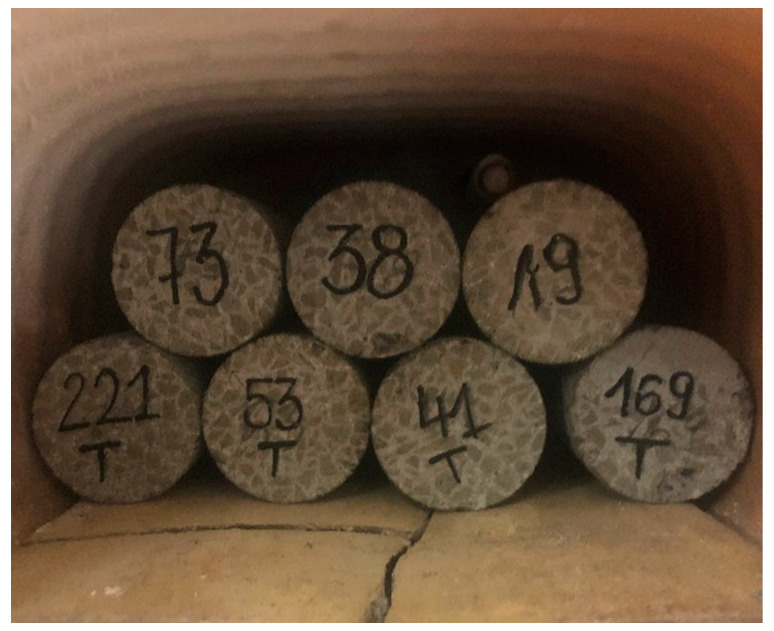
Sample arrangement during heating.

The samples were placed in a precision furnace chamber. The temperature function was determined according to [42]. Once the furnace was started up, the temperature increased by 0.5 °C/min. The target components were heated to temperatures of 200, 400 and 600 °C. Once the target temperature was reached, it was maintained for one hour in order to obtain uniform heat energy throughout the sample volume. Samples from all four mixtures were heated to the preset temperatures. After cooling, the samples were subjected to further tests. A series of samples in the furnace before heating is shown in Figure 1.

### 2.3. Testing of Concretes

Preliminary tests were carried out to determine the general properties and to check the homogeneity of the test pool. Using an electronic calliper with an accuracy of 0.01 mm, the height was determined and the diameters measured at three different locations. All cylinders were weighed on an electronic balance with an accuracy of 0.02 g. An ultrasound test was also used as part of the internal structure assessment. Ultrasound testing makes it possible to determine the degree of deterioration and the strength of the concrete [43]. A pundit lab proceq device (Schwerzenbach, Switzerland, 2013) was used for the study. Measurements were taken on samples before and after fire testing. Wave propagation velocities were determined for each sample based on the measured height and the time elapsed between the sending and receiving of the signal by the device elements.

### 2.4. Uniaxial Compression Test

Relevant to the testing process were strength tests. From each group (HPC, HPFRC 0.6, HPFRC 0.9 and HPFRC 1.1), five specimens were subjected to a compression test. The specimens were cylindrical with a diameter of 50 mm and a height of approximately 100 mm. The uniaxial compression test was carried out on a walter + bai ag servohydraulic press. Geometrical information about the sample was entered into the control software each time. The specimen was placed inside the test chamber on centering plates. The loading rate was determined as the resulting cross-sectional area of the specimen at 0.5 MPa/s.

### 2.5. Brazilian Split Tensile Test

**Figure 2:**
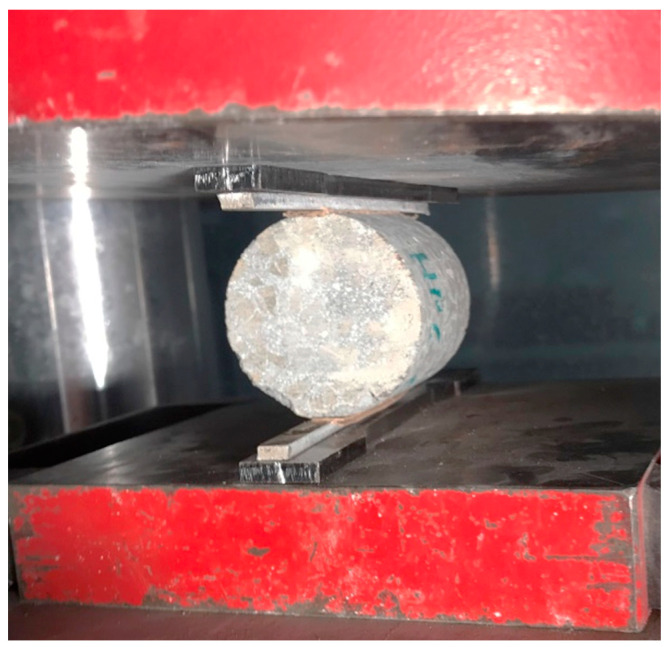
Test scheme for concrete—tension by splitting.

The test was performed in accordance with EN 12390-6:2009 on cylindrical specimens. The cylindrical samples had dimensions of approximately 50 mm (diameter) × 50 mm (height). The rate of load increase was determined in accordance with Section 6.3 of the standard. The parameter was taken as 0.05 MPa/s. Five specimens from each group and temperature were tested. Figure 2 shows a schematic of the laboratory tool inducing the tensile effect. A deviation from the standard was applied when converting the resulting forces into strength. The standard recommends determining the strength as a conversion of the force to the area of a rectangle with sides equal to the lengths of the diameter and the length of the specimen with an additional factor of 2/π. Figure 2 shows a schematic representation of the test specimen. The green colour shows the theoretical failure surface course, while the purple colour indicates the actual surface course on one of the specimens. The analysis of the differences in the surface course is one element of the study.

### 2.6. Computed Tomography

**Figure 3:**
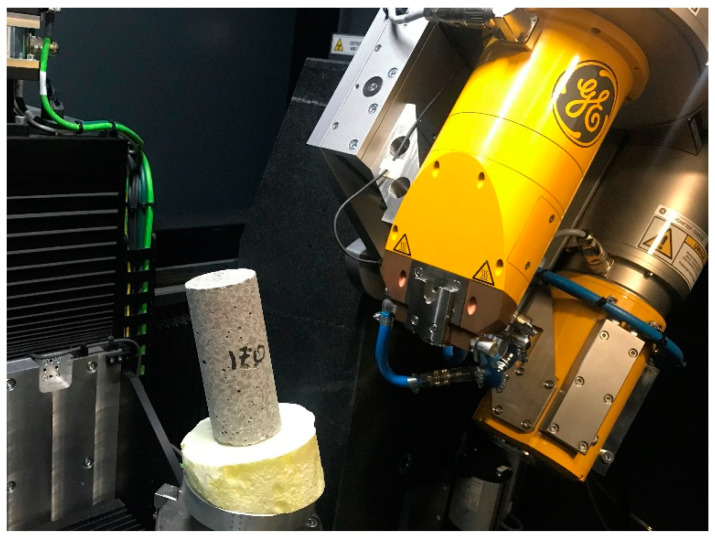
CT scanning.

X-ray tomographs (XCT, µCT, X-ray CT) for industrial use are operated on X-ray spectroscopy. A precise overview is provided in the publication [44] and despite the passage of almost 25 years, the principle of the equipment has not changed. The object is inspected in the device’s insulated chamber. The item is positioned between a radiation emitter, the so-called lamp, and the detector. Control in the scanning process is performed by determining the position of the object in the space between the lamp and the detector, setting the value of the voltage and current generating the radiation (power) and specifying the operating characteristics of the detector. Based on the operator’s experience, the selection of scanning details allows 2D projections to be obtained to enable further 3D reconstruction of the object. A single scan lasts 20–90 min. During this time, the sample rotates 360° with the vertical axis. A view of the test chamber is shown in Figure 3.

After scanning, the 3D volume should be reconstructed. During the measurement, the device saves the results as high-grey scale images (e.g., GE Phoenix v|tomex|m uses 14 bit). To improve the quality of the reconstructed data, a number of filters are used, such as beam-hardening correction, automatic geometry calibration and geometry optimisation [45,46]. The reconstructed object can be visually inspected by analysing 2D cross-sectional images, 3D images and further volumetric analysis.

Tomographic scanning has already been used in concretes to characterise the surface [47]. A detailed description of the research methodology and the possibilities in working with the reconstructed data is included in the paper [48]. Attempts have also been made towards imaging concrete under load. In the paper [49], studies on changes from static loading are presented and in [50], sheared elements were imaged. There are studies that show how to determine the change in pore volume of a concrete sample subjected to freezing cycles by tomography using a medical device [51]. Tomography allows good detection of reinforcement and helps in bonding studies [52,53]. The use of computed tomography to verify the mechanism of bond failure in fibre-reinforcement concrete is shown in [54].

It should be noted that all measurements took place with exactly the same detector settings, voltage and manoeuvring table position. The results obtained were based on reconstruction using the same algorithm: bhc + filter (factor 6.4), auto agc and reconstruction optimisation were applied. Beams were emitted with 185 kV and 160 µA characteristics.

The cylindrical samples had dimensions of approximately 50 mm × 100 mm. The assumption of maximum measurement accuracy was related to limitations from the detector size. Achieving the best accuracy is related to adjusting the position in the test chamber to a smaller dimension—the width. Due to the sample dimension, the authors used the multiscan procedure. Three scans were taken to reconstruct one sample. The first scan covered the lower part of the specimen, the second scan focused on the middle zone and the last scan focused on the upper section. The three scans were overlaid and combined. The sample was positioned so that the voxel size was 33 µm. The two reconstructed data sets (before and after heating) are brought by an algorithm developed by the authors to a common coordinate system. Once the process is complete, it is possible to fully evaluate the data under conditions as if the samples were virtually in the same place.

### 2.7. Volumetric Analysis

**Figure 4:**
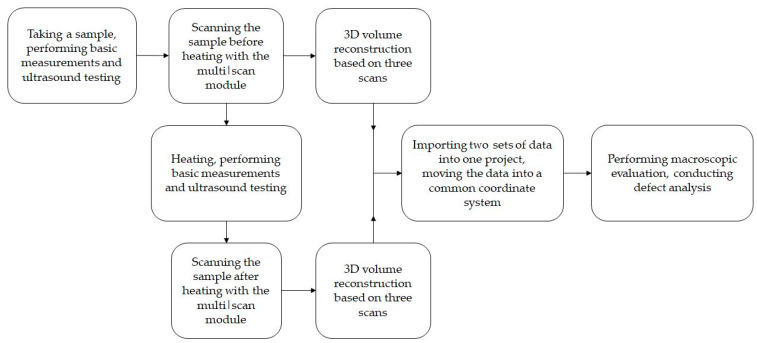
Data workflow.

The representation of the change in defects (porosity) is widely used to describe structural changes in concretes [55,56] or to identify differences that occur under the influence of external factors [54]. Commercially offered software largely automates porosity/inclusion analysis. The analysis is performed on a 3D volume. The absorption limits for concrete and air are indicated before calculations are carried out. The VGDefX algorithm in Voids mode was used. Significant pores were considered to be those whose volume exceeded 4 voxels. The analysis indicates defects in the entire data set or only in a user-specified section. The assessment is done visually and through tabular summaries and graphs. Depending on the size of the void, the pores are visualised in different colours on the sections and in the 3D view. A schematic of the workflow with the data is shown in Figure 4.

**Figure 5:**
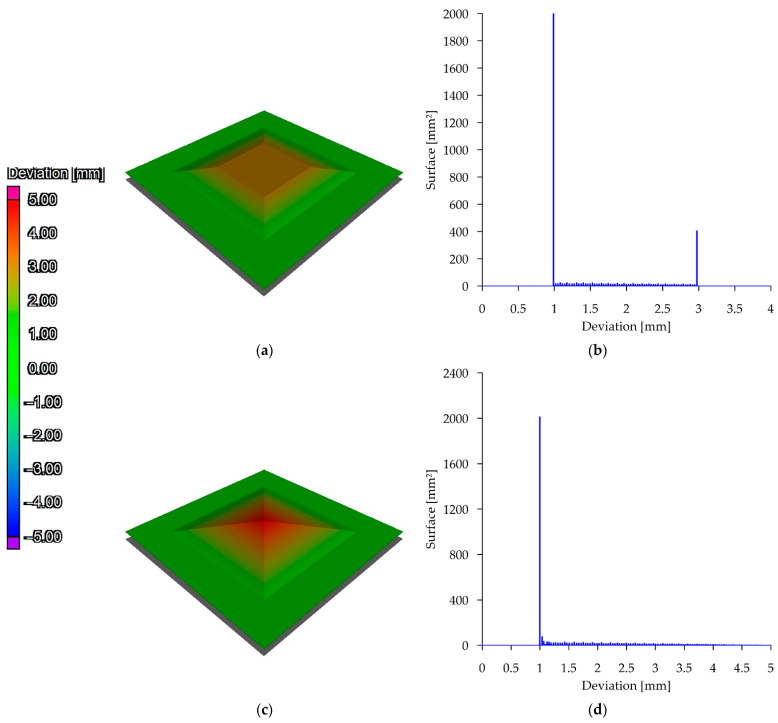
Deviation analysis methodology: (**a**) model with trapezoidal elevation; (**b**) deviation diagram; (**c**) model with triangular elevation; (**d**) deviation diagram.

The work uses a nomina/actual comparison analysis. The analysis makes it possible to determine geometric deviations between two surfaces or solids. The prerequisite for a successful analysis is that the elements are placed in a common coordinate system. Figure 5 shows the results of two example analyses. Two surfaces were introduced into the analysis environment. The nominal surface in both cases is the plane (marked grey in the figures). In the case shown in Figure 5a, there is a surface 1 mm away from the nominal surface, which additionally has a trapezoidal elevation. The object at its highest position is 3 mm away from the nominal plane. In the second case (Figure 5c), the comparison surface has an additional elevation with a triangular cross-section. The peak of the elevation is at a distance of 4 mm from the nominal surface. In their discussion, the authors refer to the interpretation of visual images and deviation histograms. According to the scale, the green colour describes the parts of the surface up to 1 mm away from the nominal object. Moving through orange to red, fragments away from the nominal plane up to 5 mm are represented. In the case of closed mesh, it is possible that the object to be analysed penetrates into the nominal object. In this case, the colours would pass through blue towards purple. The graphs shown beside on the horizontal axis show the magnitude of the deviation. The vertical axis indicates how much of the surface is at a given distance from the nominal plane. In the case of the graph describing the deviation of the first surface, two main peaks are observable—at a value of 1 mm and at a value of 3 mm. In the case of the second surface, the main peak becomes apparent at a value of 1 mm.

## 3. Results

### 3.1. Basic Tests

**Figure 6:**
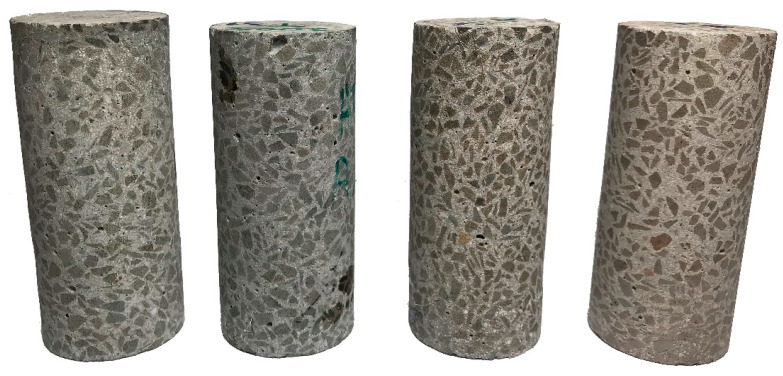
HPFRC 1.1 samples. From left: without heating, 200 °C, 400 °C, 600 °C.

Example samples are shown in Figure 6. The heating process was marked by a visual change in the appearance of the sample. No changes in the form of spalling or material separation were noted. Manual measurements (calliper) did not indicate changes in the dimensions. The dimensions were further checked during the volumetric analyses of the data acquired by CT. However, the colour of the aggregate changed. A slight colour change could already be seen at 200 °C. The aggregate darkened in the macroscopic evaluation. At a threshold of 400 °C, the 2–8 mm aggregate had taken on darker colours. Individual elements turned to orange shades. At 600 °C, there was further propagation of colour changes. Extensive research related to the colourimetry of concretes subjected to high temperatures is presented in paper [31], in which analogous results were obtained.

**Figure 7:**
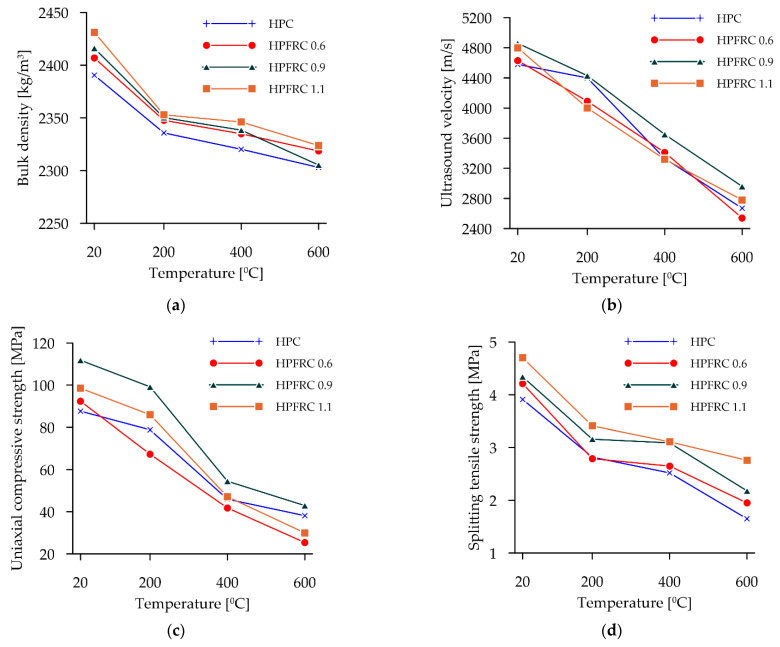
Basic test results: (**a**) bulk density; (**b**) ultrasound wave velocity; (**c**) uniaxal compressive strength; (**d**) tensile strength.

Figure 7 shows the results of the basic tests carried out for the reference samples and those subjected to high temperatures.

Figure 7a shows the variation in the volumetric (bulk) densities for all groups of concretes tested. The basic density for HPC without fibres averaged 2391 kg/m^3^. Adding fibres increased the volumetric density by 0.7% for HPFRC 0.6, 1.1% for HPFRC 0.9 and 1.7% for HPFRC 1.1. Heating each time led to decreases in the mass of the specimens and thus in the volumetric density of the material. In the case of specimens without the additional fibre component, its weights decreased by an average of 2.3% during heating at 200 °C. Samples without fibres subjected to a thermal treatment of 400 °C lost an average of 2.9% in weight. Samples subjected to 600 °C heating lost an average of 3.7% of their mass. The trend of weight loss increased when additional fibres were added to the sample. In mixtures labelled HPFRC 0.6, the mass decreased by an average of 2.5% during heating at 200 °C. At 400 °C, a weight loss of 3% was observed and HPFRC 0.6 samples subjected to heating at 600 °C lost 3.7% in weight. Samples with 0.9 kg/m^3^ lost 2.7%, 3.2% and 4.6% in weight during heating and HPC 1.1 samples lost 3.22%, 3.50% and 4.42% for heating at 200, 400 and 600 °C, respectively.

The ultrasonic results are summarised in Figure 7b. Adding polypropylene fibres to the mixtures increases the speed of wave propagation in the medium. The highest velocities were obtained at a dosage of 0.9 kg/m^3^. It was shown that heating increases the time between sending and receiving the wave, which has the effect of lowering the propagation velocity. The greatest differences between groups of specimens are observed with heating at 200 °C. For concretes without fibres, the decrease reaches 4%. For concretes with a fibre addition, the average decrease in the wave velocity was 11.7%, 8.8% and 16.7 for the HPFRC 0.6, HPFRC 0.9 and HPFRC 1.1 groups, respectively. Fibre addition is strongly associated with changes in the internal structure of the specimen, which is affected by temperatures up to 200 °C. The authors claim that this is related to the deformation temperature of the fibres, which was set at 145 °C, according to the manufacturer. When heating at 400 °C, all samples showed a similar percentage decrease in wave propagation velocity (27.9%—HPC, 26.3%—HPFRC 0.6, 24.9%—HPFR 0.9, 30.8%—HPFRC 1.1). When firing at 600 °C, further decreases in wave transition velocities were noted. The control group showed a 41.7% decrease in wave velocity, the HPFRC 0.6 group showed a 45.1% decrease, HPFRC 0.9 showed a 39.1% decrease and HPFRC 1.1 showed a 42.1% decrease. The authors note that the largest decreases in the wave propagation velocity are seen for samples with the manufacturer’s suggested fibre dosage. For the dosage used, the proportion for which the best ultrasonic results were obtained is 0.9 kg/m^3^.

The graph in Figure 7c shows the average result for the residual uniaxial compressive strength of samples subjected to thermal effects.

The starting HPC samples showed very low strength decreases. During heating at 200 °C, an average strength decrease of 10% was recorded, while samples with fibres lost 27%, 11% and 12% for HPFRC 0.6, HPFRC 0.9 and HPFRC 1.1, respectively. During heating to 400 °C, the greatest strength decreases occurred in HPFRC 1.1 (52% decrease relative to unheated samples) and HPFRC 0.9 (51% strength decrease). HPFRC 0.6 specimens decreased their uniaxial compression strength properties by 55%, and the non-fibre specimens by 47%. For the samples, without fibres, which were subjected to thermal exposure up to 600 °C, the downward trend continued and amounted to 56% relative to the non-heated samples. For samples with fibres, the decrease was 72% for HPFRC 0.6, 62% for HPFRC 0.9 and 70% for HPFRC 1.1. The highest strength after the entire test cycle was obtained for HPFRC 0.9 samples (42.92 MPa).

The results of tensile tests by splitting according to EN 12390-6:2009 are shown in Figure 7d. HPC samples lose strength in a quasi-linear manner—a decrease of 28% for 200 °C, 35% for 400 °C and 58% for 600 °C. The loss of properties for the fibre-reinforcement samples followed a similar pattern. A large loss of properties occurred when the samples were heated to 200 °C (a decrease of about 34% for all groups). When heated to 400 °C, HPFRC 0.9 and HPFRC 1.1 samples reached a similar strength of 3.1 MPa (a decrease of 29% for HPFRC 0.9 and 34% for HPFRC 1.1, relative to the non-heated samples). Finally, at 600 °C, HPFRC 0.6 and HPFRC 0.9 samples lost 54% and 50% of their tensile strength, respectively, while HPFRC 1.1 samples lost 41%.

### 3.2. Results of the Destruction Surface from the Splitting Tensile Test

Due to the observed strong influence of both the amount of polypropylene fibres and the effect of the heating temperature on the basic physico-mechanical parameters of the concretes tested, this paper addresses the quantitative description of the change in the structure of the concretes tested and its effect on the failure surface in the splitting tensile test.

**Figure 8:**
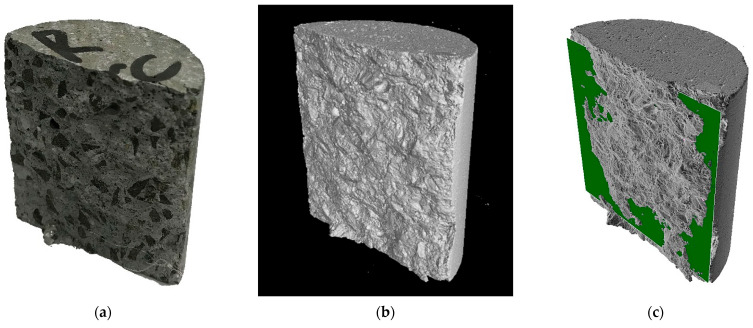
Tensile by splitting: (**a**) Post-test specimen; (**b**) CT-based reconstructed model; (**c**) Specimen including nominal plane.

**Figure 9:**
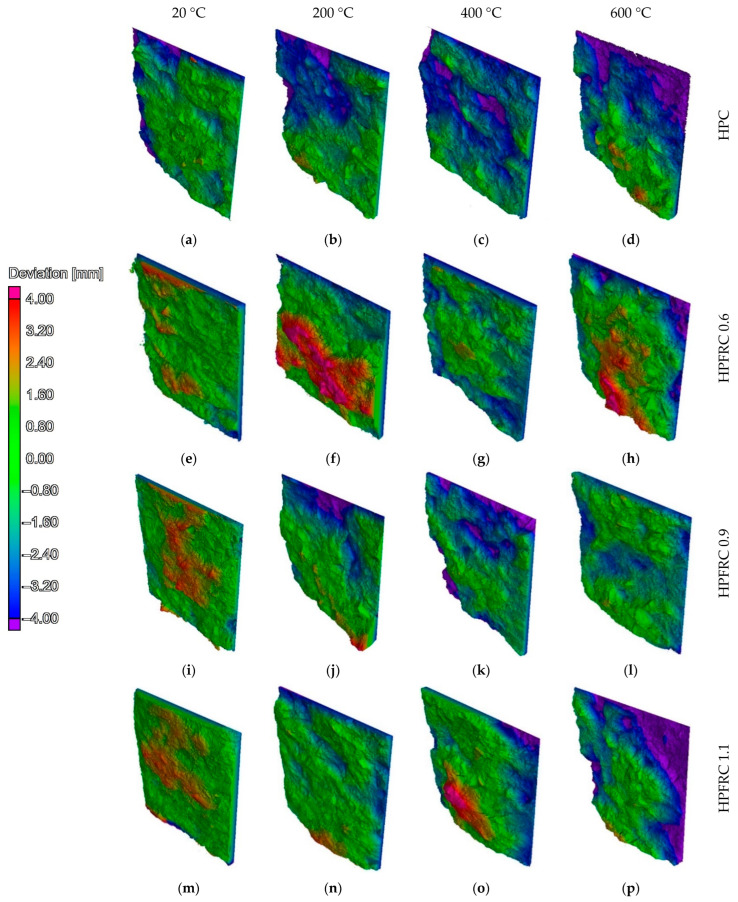
Map of surface deviations after tensile by splitting tests from a hypothetical plane based on vertical diameter: (**a**) HPC 20 °C; (**b**) HPC 200 °C; (**c**) HPC 400 °C; (**d**) HPC 600 °C; (**e**) HPFRC 0.6 20 °C; (**f**) HPFRC 0.6 200 °C; (**g**) HPFRC 0.6 400 °C; (**h**) HPFRC 0.6 600 °C; (**i**) HPFRC 0.9 20 °C; (**j**) HPFRC 0.9 200 °C; (**k**) HPFRC 0.9 400 °C; (**l**) HPFRC 0.9 600 °C; (**m**) HPFRC 1.1 20 °C; (**n**) HPFRC 1.1 200 °C; (**o**) HPFRC 1.1 400 °C; (**p**) HPFRC 1.1 600 °C.

A photo of an example specimen after a tensile test is shown in Figure 8a. Figure 8b shows the reconstruction result of the tomographic scan data. The authors note the variation of the splitting surface in tensile tests. The actual surface is never a plane. The maps of the resulting failure surfaces in Figure 9 show examples of the geometric deviations of the actual plane obtained in the splitting tensile tests relative to the plane passing vertically through the centre of the specimen shown in Figure 8c.

**Figure 10:**
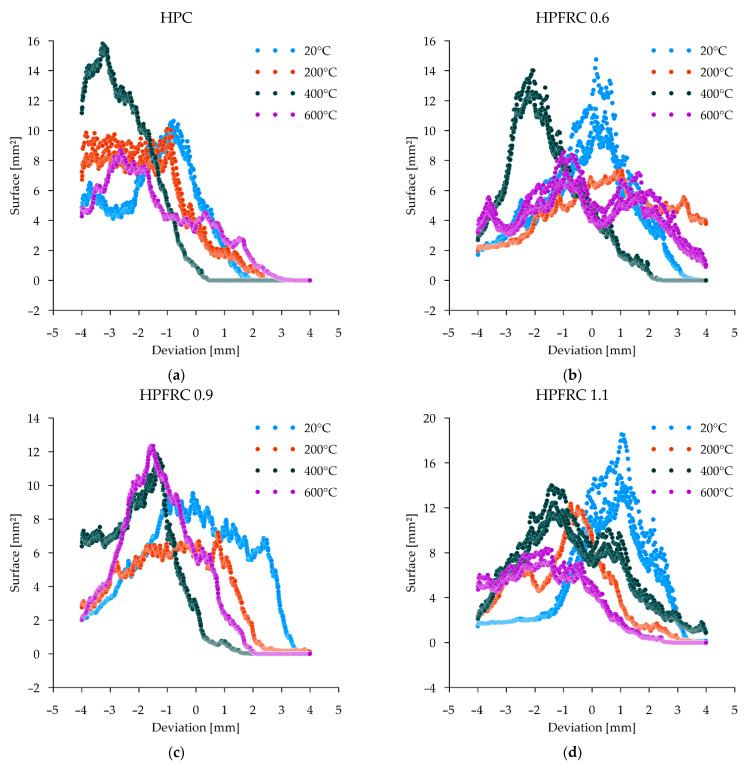
Deviation histograms: (**a**) HPC; (**b**) HPFRC 0.6; (**c**) HPFRC 0.9; (**d**) HPFRC 1.1.

Figure 10 shows the results of the failure surface shape analysis obtained in the splitting tensile test. The reference specimens were characterised by a low level of deviation from the nominal planes (peaks of the graphs close to deviation = 0). The graphs confirm that deviations tend to concentrate around a single peak, gradually reducing the values of the fields assigned to a given distance. At 20 °C, attention is drawn to the very small number of places where deviations greater than 4 mm occur. In the case of HPFRC 0.9 and HPFRC 1.1 specimens, a slight change in the shape of the failure surface towards an increase in inhomogeneity can be observed (deviations in the range −2 to 3 mm). With an increase in temperature up to 200 °C, the surface becomes rougher, as shown in the diagrams by the flattening and elongation of the peaks. In the case of specimens heated to 400 °C and 600 °C, failure occurred, among other things, through the detachment of the aggregate from the matrix. Deviations of more than 4 mm from the nominal plane were observed over large areas, corresponding to half the diameter of the coarse aggregate dimension.

**Figure 11:**
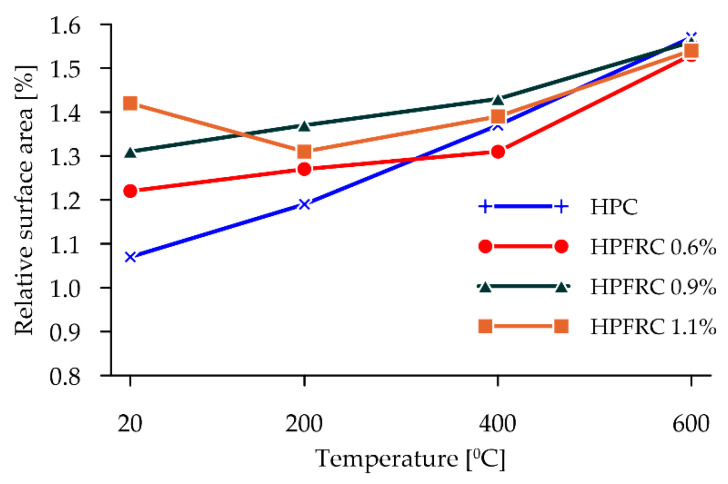
Effect of heating temperature on failure surfaces.

Confirmation of the observed phenomena related to the development of the shape of the post-failure surface as a result of the splitting tensile test is provided by the results of calculating the relative surfaces, i.e., the ratio of the reference plane to the specified failure surface by CT scanning (Figure 11).

The results clearly show an increase in the relative failure area for unheated concretes in proportion to the number of fibres used. This influence increases with the increasing heating temperature, while the influence of the proportion of polypropylene fibres alone resulting from their melting disappears.

### 3.3. Computed Tomography

**Figure 12:**
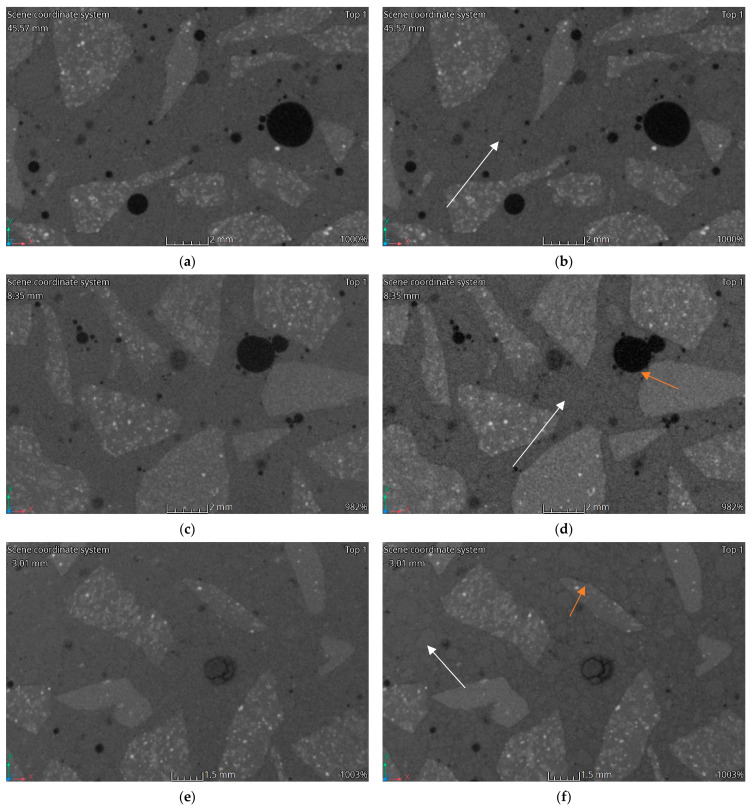
CT imaging results. Structural changes in three samples HPC: (**a**) 20 °C; (**b**) 200 °C; (**c**) 20 °C; (**d**) 400 °C; (**e**) 20 °C; (**f**) 600 °C.

**Figure 13:**
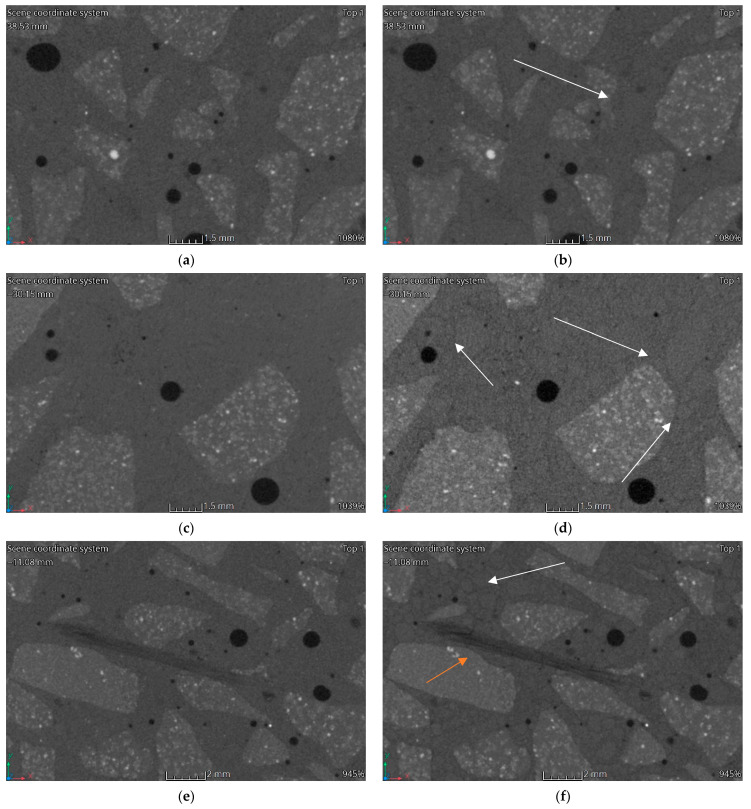
CT imaging results. Structural changes in three samples HPFRC 0.6: (**a**) 20 °C; (**b**) 200 °C; (**c**) 20 °C; (**d**) 400 °C; (**e**) 20 °C; (**f**) 600 °C.

**Figure 14:**
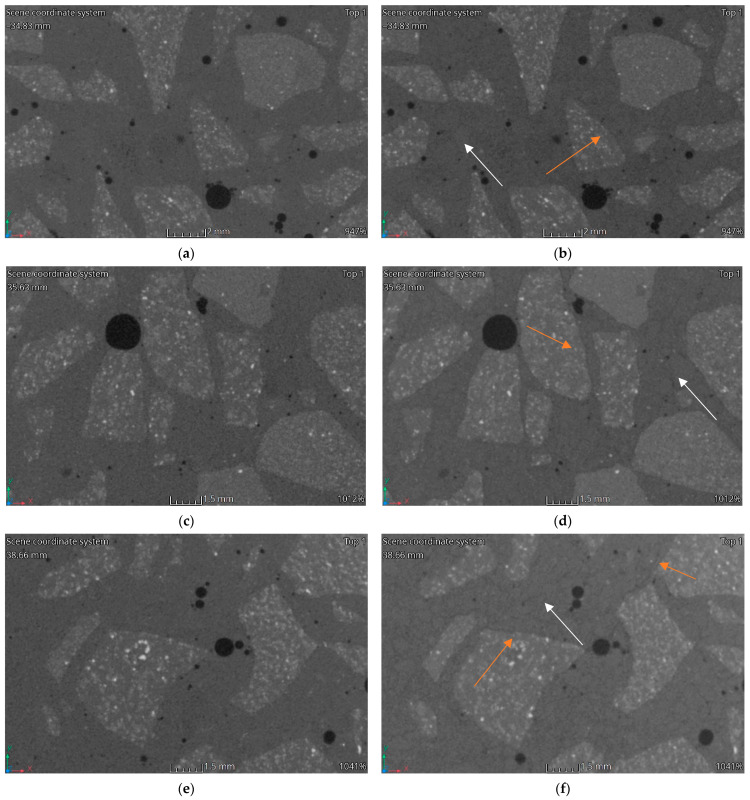
CT imaging results. Structural changes in three samples HPFRC 0.9: (**a**) 20 °C; (**b**) 200 °C; (**c**) 20 °C; (**d**) 400 °C; (**e**) 20 °C; (**f**) 600 °C.

**Figure 15:**
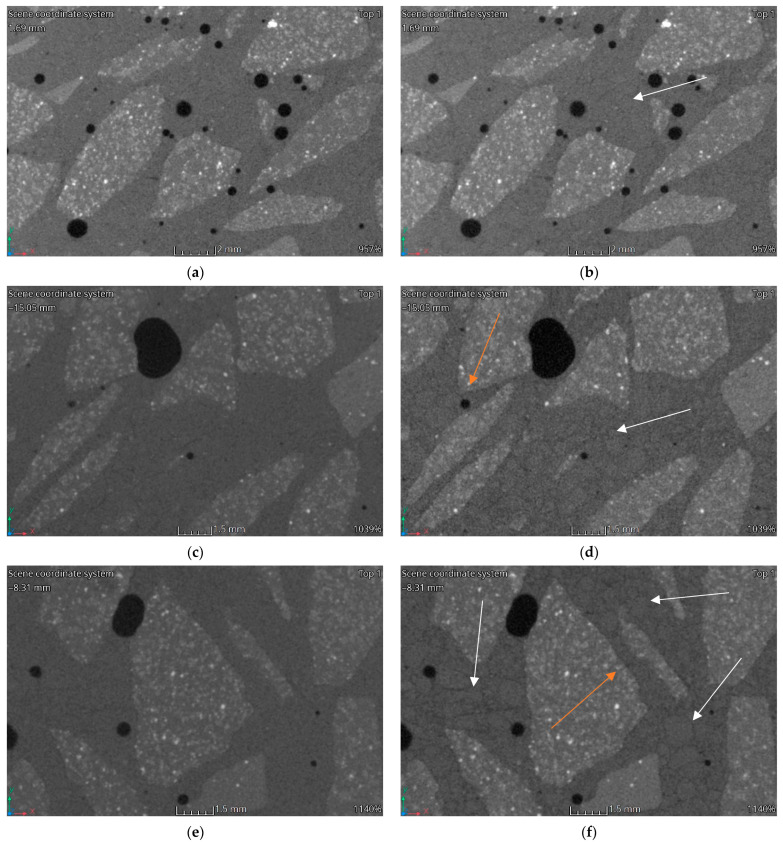
CT imaging results. Structural changes in three samples HPFRC 1.1: (**a**) 20 °C; (**b**) 200 °C; (**c**) 20 °C; (**d**) 400 °C; (**e**) 20 °C; (**f**) 600 °C.

Figure 12, Figure 13, Figure 14 and Figure 15 show vertical cross-sections of tomographically scanned samples. The samples are grouped with respect to the mixture. Side-by-side views are shown before and after heating at a specific temperature (200 °C, 400 °C and 600 °C, respectively). Visually noticeable are the changes in the internal structure at 400 °C and 600 °C. The colour change mainly concerns the matrix—the interpretation of the results indicates a change in the material density. A change to a darker colour proves a decrease in the density of the matrix material. By increasing the heating temperature, the changes become more evident.

During heating, the fine aggregate gradually becomes visible in the matrix. Visually, it stands out through the formation of a dark outline. The dark colour is associated with a local reduction in the density of the material. The authors interpret this phenomenon as a weakening of the adhesion between the aggregate and matrix and, at higher temperatures, the formation of micro-cracks. In Figure 12, Figure 13, Figure 14 and Figure 15, areas with changes around the fine aggregate are marked with white arrows. A second observation is the formation of air gaps between the aggregate 2–8 and the matrix. These changes are visible in all groups of samples at temperatures above 400 °C. In Figure 12, Figure 13, Figure 14 and Figure 15, areas with changes around aggregate 2–8 are marked with orange arrows.

### 3.4. Defect Analysis

**Figure 16:**
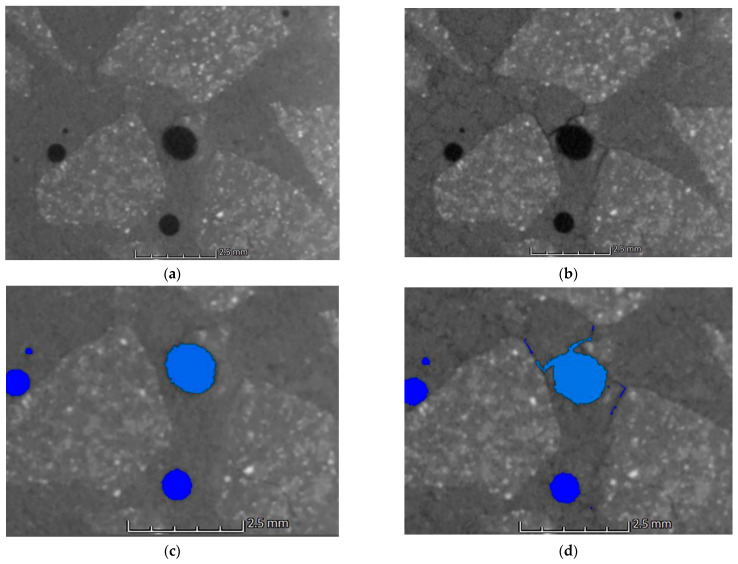
Porosity of HPFRC 1.1 sample heating to 600 °C: (**a**) before heating; (**b**) after heating; (**c**) graphical result of analysis before heating; (**d**) graphical result of analysis after heating.

Defect analysis was carried out on the reconstructed X-ray computed tomography data. Figure 16 shows a section of a specimen made from the HPFRC 1.1 mixture fired at 600 °C. The images show the same air pore with an average diameter of 1.18 mm. The sample, before exposure, shows good matrix quality and the air pores do not isolate the aggregate from the matrix. The pores are characterised by high sphericity. Figure 16b shows the same cross-section of the sample after the thermal impact. In addition to the pores, numerous cracks are visible all around. The crack surfaces run between the aggregates. The sample is clearly weakened. In addition, 0–2 mm sand grains have become distinguishable. Zones of a significantly lower density appeared around the grains of the finest aggregate. These zones can be interpreted as micro-cracks at the grain–matrix contact. Defect analysis was carried out to detect air voids before and after heating on the reconstructed 3D volumes. Visualisation of the analysis results is shown in Figure 16c (before heating) and Figure 16d (after heating). Changes in the form of marked cracks were included in the total defect volume. The analysis was carried out on parameters selected for the entire test group, allowing the authors to compare the results of all groups together with changes at all temperatures.

Table 2 summarises the porosity of the analysed cylindrical specimens before and after heating, obtained by volumetric analysis on the reconstructed volumes of the scanned specimens. The highest porosity was that of the HPC, noting that this may be due to the fact that more superplasticiser was applied to the HPFRC mixtures to increase their workability, resulting in a reduction in the porosity of these concretes as a result of their improved compaction.

**Figure 17:**
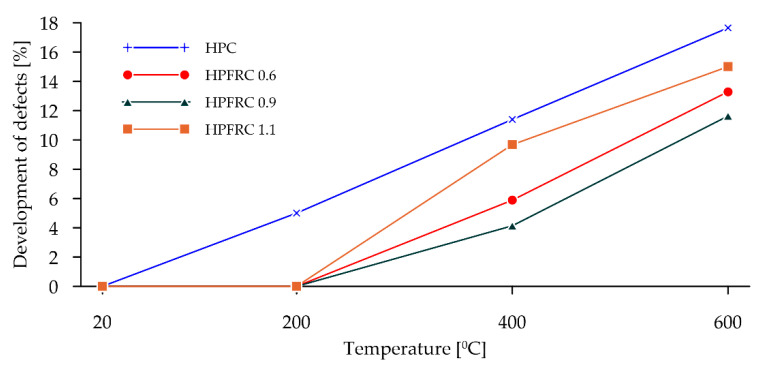
Defects development.

The percentage change in the porosity of each group of specimens relative to the volume of pores in the specimens before heating is shown in Figure 17. It is noticeable that there was no increase in the porosity of the specimens with fibres heated up to 200 °C, whereas for specimens without polypropylene fibres, the porosity increased by 5% relative to the volume before firing. The smallest increases in porosity were observed in samples with 0.9 kg/m^3^ polypropylene fibres. The largest increase in porosity was observed in samples without fibres.

## 4. Discussion

The decrease in the density of concrete specimens without polypropylene fibres had a non-linear course. These results are in line with the literature data presented, among others, in the paper [57], in which, for tests on lightweight concretes, the greatest drop in density was obtained when the specimens were heated to 200 °C, while the value of the drop decreased when the specimens were heated at 400 °C and 600 °C. The decrease in the volumetric density is related to the chemical processes taking place in the cement matrix, as a result of which, among other things, water evaporation takes place and the first micro-cracks can already form.

For the specimens containing polypropylene fibres, similar phenomena were observed, but it is worth noting that, initially, the specimens containing polypropylene had a higher density in proportion to the fibre content. This is related to the increase in the workability of the mixture under the influence of the superplasticiser used, which resulted in a reduction in the porosity of these concretes due to their better compaction. The recorded decreases in density for a heating temperature of 200 °C are analogous to those in the paper [58]. It can, therefore, be concluded that up to 200 °C, the decrease in density is mainly due to the melting of the polypropylene, which is partially absorbed by the cement matrix. At 400 °C and 600 °C, the fibres have fully burnt down, transforming into CO_2_ and H_2_O, and therefore, the smaller decrease in density for these temperatures is due to the formation of micro-cracks in the structure of the concretes analysed. It should be noted that at 360–400 °C, molten polypropylene releases (e.g., pentane, propylene) [59], which can further impact on pore pressure.

The change in density with an increasing heating temperature is also reflected in a decrease in the ultrasonic wave transition velocity. This change can be described as linear especially for concretes containing polypropylene fibres. In the case of HPC without polypropylene fibres, a higher change is only observed when the specimens are heated above 200 °C. When ultrasonic waves pass through concrete and run into defects (micro- and macro-cracks), their original propagation path and mode of propagation is affected, which is usually accompanied by a decrease in the ultrasonic pulse velocity [43]. In the case of HPCs, due to the lack of polypropylene fibres, the first micro-cracks are already formed in the temperature range of 20–200 °C and, in fact, beyond 100 °C, i.e., at the point at which water changes from a liquid to a gas. This causes an increase in pressure in the micropores and, consequently, micro-damage to the concrete. On the other hand, in the case of concretes containing fibres in the temperature range analysed, the fibres burn out, which increases the number of pores in the specimens tested [60] and thus causes a decrease in the wave transition velocity. It is worth noting that analysis of the decrease in the ultrasonic wave transition velocity may indicate that the volume of micro-cracks in HPC heated to 200 °C is smaller than the volume of fired polypropylene fibres in the other concretes (a percentage decrease in the wave transition velocity is observed to be lower in HPC than in HPFRC). However, this is not confirmed by the results of the volumetric density alone of the concretes tested. It is also worth noting that the decreases in ultrasonic wave transition velocity for the HPCs and HPFRCs analysed are smaller than those registered for normal concretes [61].

The addition of polypropylene fibres changed the rheological properties of the mix, resulting in the need for more superplasticiser. As a consequence, this led to an increase in the volumetric density of the concrete and a strengthening of its structure, resulting in an increase in compressive strength. However, it should be noted that this increase depended on the amount of fibre used. In the study conducted, the best results were obtained for the addition of polypropylene fibres at 0.9 kg/m^3^ of concrete mix. The influence of the effect of the quantity and size of the fibres has been extensively described, among other, in papers [62,63].

**Figure 18:**
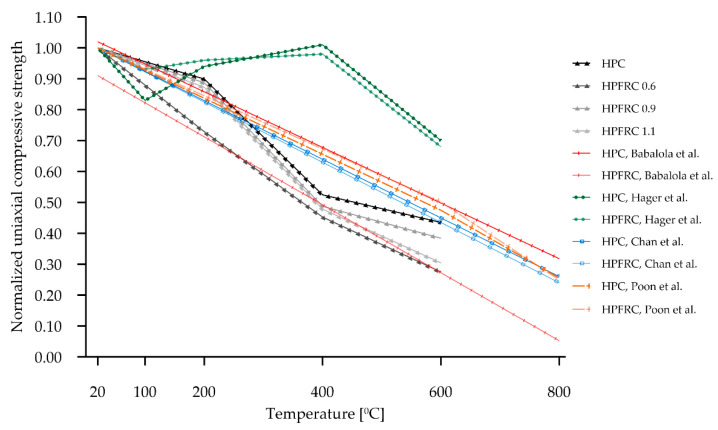
Comparison of residual compressive strength with literature data [35,36,37,38].

As the specimens are heated at increasingly higher temperatures, the residual compressive strength of high-value concretes generally decreases in a linear or quasi-linear manner. This is related to the dehydration of the CSH gel, the decomposition of the portlandite and the destruction of the contact zone due to the different thermal expansion coefficients of the cement matrix and the aggregate. Figure 18 summarises the results obtained for HPC and HPFRC (black and grey) compared to the literature data.

In paper [36] (Figure 18, green), there was a significant decrease in the compressive strength for concretes heated to 120 °C, followed by an increase in this strength for concretes heated to 200 °C. This increase did not exceed the initial strengths determined at 20 °C and remained until about 400 °C and then the residual compressive strength decreased rapidly. This initial strength decrease was found to be related to the presence of a moisture gradient in the concrete specimens. In HPFRC, these phenomena had a softer course than in HPC. In paper [37], the effect of temperature was investigated in the range of 20 °C and 800 °C, and in this range, a minimal effect of fibres on the residual strength of the concrete was noted. Very similar results were obtained in work [38] for testing at 400 °C and 600 °C. In paper [35], the authors carried out a comprehensive review of concretes exposed to high temperatures and the influence of various concrete additives on these results. For selected papers, they derived correlated linear relationships for normalised residual compressive strength over the temperature increase range of 0 to 1000 °C. These are shown in red in Figure 18, but it is worth noting that, despite the high coefficient of determination of these correlations, the description by a linear function will not always be accurate (this can be seen for HPFRC where the initial value of the normalised residual compressive strength for t = 20 °C is 0.91). However, the authors note that the most interesting phenomena, in the context of the polypropylene fibre values, occur between 20 and 200 °C and that there are relatively few papers presenting them so far.

**Figure 19:**
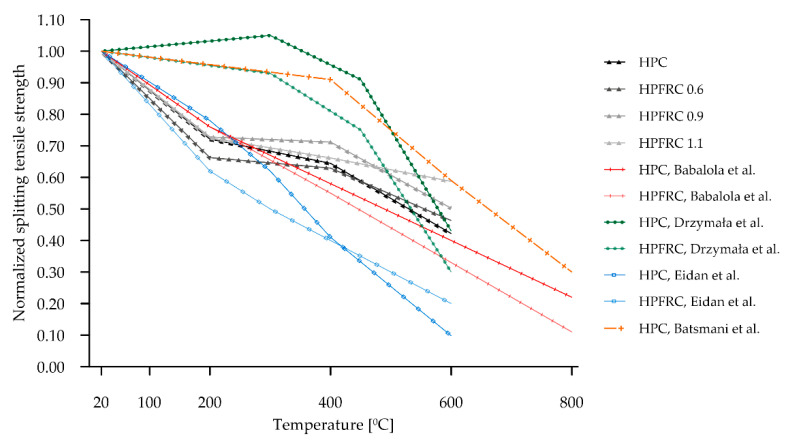
Comparison of tensile strength with literature data [17,35,39,65].

As with compressive strength, the residual splitting tensile strength decreases with the increasing temperature. It is worth noting that a reference to this parameter is much rarer in the literature, as stated, for example, in paper [64]. In general, studies on the effect of polypropylene fibres on concrete indicate that their addition in appropriate proportions increases the tensile by splitting strength, but this effect is not persistent with temperature exposure. This is due in part to the low temperature resistance of polypropylene plastic. The effect of temperature on the decrease in this characteristic is greater than in compression, which is due to the nature of the damage that occurs in compression and tension: in compression, micropores and micro-cracks will tend to tighten first, while in tension, they will open up, causing them to propagate and accelerate the destruction of the structure [64]. Figure 19 summarises the results of the normalised residual tensile by splitting strength obtained in the present study (black and grey) against the literature data.

In paper [17], there was a slight increase in the tensile strength for HPC in the heating range up to 300 °C and a slight decrease in this range for HPFRC. Beyond this temperature, a sharp decrease in tensile strength is evident. Similar results were obtained in work [65], in which only the HPC was investigated. The other works show a much greater downward trend already at heating temperatures up to 200 °C. In paper [39], different HPFRC mixes were tested—HPC reference concrete and HPFRC containing 6 mm polypropylene fibres at 1 kg/m^3^ were selected for comparison. In this work, a quasi-linear decrease in strength was obtained, with initially greater decreases recorded for the HPFRC tests, then the trend changes and finally, for 600 °C, a greater decrease was recorded for the concrete without fibres than for the concrete with fibres. As in the case of compressive strength, the authors in [35] presented linear relationships (red in Figure 19). These results should be regarded as average values from tests with different mix compositions and seem to fit well with the trends presented. In the present study, in the first temperature range (0–200 °C), the decrease obtained is relatively high (comparable to that in the work: [35,39]), In the 200–400 °C range, the course of this decline is closer to the work of the [17,65]. In the last range analysed, i.e., above 400 °C, all works show significant drops in residual strength.

The phenomena described regarding changes in the strength parameters and volumetric density are confirmed by porosity analysis of the concretes tested and CT analysis. It should be noted that in all HPFRCs, no increase in porosity was observed up to the heating temperature of 200 °C. No significant changes around the individual concrete components were registered in the CT images Figure 13b, Figure 14b and Figure 15b). It is only above this temperature that changes are visible around both the coarse and fine aggregate, with zones of a significantly lower density and clear micro-cracking at the grain–matrix contact at 600 °C (Figure 16d). The recorded zones of reduced density in the cement matrix are caused by chemical phenomena in the matrix, which are induced, among other things, by the decomposition of the Mg(OH)_2_, Ca_3_Al_2_O_6_·CaSO_4_·12H_2_O, Ca_3_Al_2_O_6_·6H_2_O and Mg_6_Al_2_O_9_·CO_3_·12H_2_O. One of the most important, due to the lower density zones recorded for temperatures above 400 °C, is the breakdown of Ca(OH)_2_. Finally, at temperatures above 500 °C, the breakdown of Al(OH)_3_, CaCO_3_ and MgCO_3_ occurs. An additional factor causing the formation of micro-cracks is the different thermal expansion of the fine and coarse aggregate grains and of the cement matrix itself

HPC in the temperature range of 20–200 °C behaved slightly differently to HPFRCs. Already in this temperature range, a 5% change in porosity relative to the pore volume was found in the samples before heating. This is mainly due to the thermo-humidity effect consisting of the occurrence of gas and liquid pressure contained in the concrete pores, accompanying the rapid evaporation of moisture from the concrete. In addition, taking into account the previously described negative effects of aggregate grains with different thermal expansions, it can be concluded that the change in the porosity of HPC at temperatures up to 200 °C is due to the formation of the first micro-cracks. This phenomenon is confirmed by the first changes around the grains of the fine aggregate recorded in CT tests (Figure 12b). This confirms the positive effect known from the literature of polypropylene fibres which, melting at 145 °C, i.e., at a temperature lower than the temperature at which the maximum water vapour pressure in the concrete occurs, are partly absorbed into the matrix and partly leave behind a network of open pores, thus reducing the internal pressure in the heated concrete, which has no destructive effect on the cement matrix. The results obtained for changes in the porosity of the concretes studied at 200 °C indicate that the HPC already showed structural changes in the matrix caused, among other things, by the formation of the first micro-cracks, while the HPFRC showed the partial melting of the polypropylene fibres. However, the resulting pore network at the temperature analysed does not yet translate into an increase in the porosity of the concrete. At temperatures of 400–600 °C, analogous phenomena occur more in HPFRCs compared to those in HPCs due to the complete melting of the polypropylene fibres. Therefore, the specific porosity increases in these temperature ranges are similar to the porosity increases recorded in HPCs.

**Figure 20:**
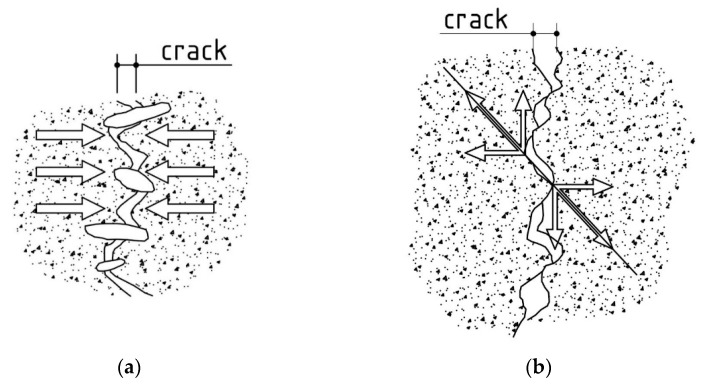
Influence of the shape of the cracking surface on the load-bearing capacity of a reinforced concrete section [67]: (**a**) bridging effect model; (**b**) aggregate interlock phenomenon model.

The results of the analysis of the failure surface in the splitting tensile test show a significant change in its shape in both HPC and concretes containing polypropylene fibres. As the amount of polypropylene fibres rises, an increase in the relative failure surface is observed in the non-heated concretes. This phenomenon is caused by the adhesion of the polypropylene fibres to the matrix, which cracks due to tensile stresses, and the embedded fibres pull out larger amounts of material compared to HPC. This results in a more varied failure surface in HPFRCs, which can have a beneficial effect on, among other things, the load-bearing capacity of cracked reinforced concrete structures resulting from the ‘bridging effect’ and ‘aggregate interlock’ phenomena. The bridging effect is illustrated in Figure 20a. When a crack appears, the aggregate grains used in the concrete mixture wedge themselves on the edges of the crack surface, thereby increasing the load-bearing capacity of the analysed section in relation to the cracked element in which this phenomenon would not occur. Of course, this phenomenon only has a positive effect on the load-bearing capacity of the section within a certain range of crack opening. “Aggregate interlock”, i.e., the “overlapping” of mutually unequal edges of the resulting crack (Figure 20b), is a term which can be used here as the mechanism of this phenomenon and the effect on the section resistance is the same as in the case of the “bridging effect”. The similarity between the two phenomena is so significant that they are very often treated as a single phenomenon in the literature and appear under the common name of an “aggregate interlock”. The shape of the resulting failure surfaces can also have an impact in the concrete composite structures by directly influencing the evaluation of the bond strength between two concrete layers [66].

As the heating temperature goes up, an increase in the relative failure area was registered, which is due to the freer detachment of larger cement matrix elements and grains of both a fine and coarse aggregate. The occurrence of micro-cracks in the contact zone between the matrix and the aggregate, as well as the weakening of the cement matrix itself, enhance the detachment process of the aggregate grains in both HPCs and HPFRCs. It should be noted in this context that, due to the melting of the polypropylene fibres at 400 °C, similar values of the relative failure area were obtained for all concretes, with a simultaneous peaking at 600 °C.

## 5. Conclusions

This paper focuses on the study of the structure of high-performance concrete with polypropylene fibre additives at different dosages subjected to temperatures of 200 °C, 400 °C and 600 °C. It should be remembered that the behaviour of high-performance concretes (HPC) under high temperature conditions can be a significant limitation in their application. As numerous studies have shown, the use of polypropylene fibres improves the durability of HPCs when exposed to high temperatures. Nevertheless, it is worth describing the phenomena and changes in the structure of such concretes due to high temperatures. The research and analyses carried out allow the following conclusions to be formed:

- The HPFRCs tested at temperatures below the melting point of the polypropylene fibres used had higher strength parameters than the reference HPC. This is due to the contribution of the fibres used in transferring the load through the adhesive forces between their surface and the cement matrix. It should be noted, however, that the best results were obtained in specimens with 0.9 kg/m^3^ of polypropylene fibres.
- HPCs and HPFRCs subjected to high temperatures reduced their strength parameters. The effect of temperature on the reduction in tensile strength is greater than in compression, due to the nature of the damage that occurs in compression and tension. In addition, especially in the case of tensile strength, it should be noted that for temperatures of 200 °C, 400 °C and 600 °C, the influence of polypropylene fibres disappears due to their complete melting. Consequently, the load-bearing capacity of HPFRCs at higher temperatures is determined by the load-bearing capacity of the other concrete components analogously to HPC.
- The positive effect of polypropylene fibres is mainly observed between 20 °C and 200 °C. The melting of polypropylene fibres causes a delay in the development of micro-cracks in the structure of these concretes compared to HPC. In connection with the above, a detailed analysis of the structural changes occurring primarily between 20 °C and 200 °C is planned in the associated studies.
- Analysis of the images obtained using the CT method confirmed the assumptions made regarding changes in the structure of HPC and HPFRC under the influence of all temperatures. The analysis of defects showed that, under the influence of high temperatures, zones of a significantly lower density appear outside the pores in the cement matrix. In addition, numerous cracks appear around the grains of both the fine and coarse aggregates, which is reflected in the porosity of the concretes tested. However, it should be noted that in the case of HPFRCs, their porosity only changes from 200 °C and above, which may indicate that the heating fibres provide free space to reduce any pore pressure, ultimately resulting in little change in the concrete structure and less change in the cement matrix itself compared to HPC.
- The CT images of the structure of HPC clearly show that the first micro-cracks appear in the concrete already at 200 °C and that this phenomenon increases in proportion to the temperature increase. This confirms the positive effects of polypropylene fibres as one method of increasing the heat resistance of concretes.

## Figures and Tables

**Table 1:** Composition by mass of a mix.

Composition		Mass [kg/m^3^]
Cement CEM I 42.5 R		550
Water		176
Sand 0–2 mm		790
Basalt aggregate 2–8 mm		940
Superplasticiser		5.14 (HPC)/7.56 (HPFRC)
Texa-Fib 3	HPFRC 0.6	0.60
	HPFRC 0.9	0.90
	HPFRC 1.1	1.10
Water/binder ratio		0.32

**Table 2:** Comparison of porosity before and after heating.

Sample		Before Heating [%]	After Heating [%]
200 °C	HPC	1.40	1.47
	HPFRC 0.6	1.17	1.17
	HPFRC 0.9	1.11	1.11
	HPFRC 1.1	0.70	0.70
400 °C	HPC	1.58	1.76
	HPFRC 0.6	1.21	1.26
	HPFRC 0.9	0.68	0.72
	HPFRC 1.1	0.61	0.67
600 °C	HPC	1.70	2.00
	HPFRC 0.6	1.43	1.62
	HPFRC 0.9	1.29	1.44
	HPFRC 1.1	0.84	0.99

## Data Availability

The data that support the findings of this study are available from the corresponding author upon request.
